# Occurrence of ‘*Candidatus* Mycoplasma haemosuis’ in fattening pigs, sows and piglets in Germany using a novel *gap*-based quantitative real-time PCR assay

**DOI:** 10.1186/s12917-022-03147-1

**Published:** 2022-01-17

**Authors:** Julia Ade, Julia Stadler, Mathias Ritzmann, Christina Zübert, Katharina Hoelzle, Ludwig E. Hoelzle

**Affiliations:** 1 Institute of Animal Science, Department of Livestock Infectiology and Environmental Hygiene, University of Hohenheim, Garbenstr. 30, 70599 Stuttgart, Germany; 2 Clinic for Swine, Centre for Clinical Veterinary Medicine, Ludwig-Maximilians-Universität Munich, Sonnenstrasse 16, 85764 Oberschleissheim, Germany

**Keywords:** Haemotrophic mycoplasmas, ‘*Candidatus* Mycoplasma haemosuis’, Quantitative PCR, Diagnosis, Vertical transmission, Pre-suckling piglets, Vertical transmission, Farrowing sows

## Abstract

**Background:**

The appearance of the novel porcine haemotrophic mycoplasma (HM) species ‘*Candidatus* Mycoplasma haemosuis’ was reported in apparently healthy but also in clinically sick animals in China, Korea and in a case report from Germany. Outside of Asia, however, nothing further is known about the frequency of ‘*Ca*. M. haemosuis’ in pigs to date. To investigate the distribution of this novel HM species in Germany, fattening pigs, sows and pre-suckling piglets were examined using a herein developed quantitative real-time PCR assay (qPCR). Because the piglets were sampled before the first colostrum uptake, additional information on a possible vertical transmission from dams to their offspring was obtained.

**Results:**

Our novel qPCR assay successfully detected ‘*Ca*. M. haemosuis’ in all blood samples from the ‘*Ca*. M. haemosuis’-infected pigs. No cross-reactivity was detected when DNA from non-target *Mycoplasma* spp. and other bacterial species representing 10^5^ bacteria/reaction were used as a template. The lower limit of detection of the qPCR was thus 10 *gap* gene copies per reaction and 2.5 x 10^3^ genome equivalents (GE) per mL blood.

‘*Candidatus* M. haemosuis’ was detected by this qPCR in blood samples from a total out of 6.25% sows (13/208), 4.50% pre-suckling piglets (28/622) and 17.50% fattening pigs (35/200). On farm level, 3 out of 21 piglet producing farms (14.28%) and 9 out of 20 fattening farms (45.00%) were positive for ‘*Ca.* M. haemosuis’. Co-infections with *M. suis* were evident in all age groups.

**Conclusion:**

‘*Candidatus* M. haemosuis’ infection is present in German pig farms and the detection of the novel porcine HM species in piglets immediately after birth before colostrum intake indicates vertical transmission. The novel qPCR assay specific for ‘*Ca.* M. haemosuis’ described herein will be a prerequisite for future studies on the prevalence, epidemiology as well as the clinical and economic impact of ‘*Ca.* M. haemosuis’ infections.

**Supplementary Information:**

The online version contains supplementary material available at 10.1186/s12917-022-03147-1.

## Background

Haemotrophic mycoplasmas (HMs) are a group of cell-wall less bacteria with a unique tropism for erythrocytes. HMs were found worldwide in a wide range of mammals, including livestock and companion animals as well as wild animals and humans [1–5]. In pigs, the two HM species *Mycoplasma suis* and *Mycoplasma parvum* were first described in the first half of the last century [6]. While *M. parvum* seems to be an apathogenic HM species, *M. suis* is the causative agent of infectious anaemia in pigs (IAP) [4, 7, 8]. Acute *M. suis* infections are characterised by haemolytic anaemia, high fever, icterus, hypoglycaemia, intravascular coagulopathy, and endothelial damages [4, 6, 9, 10]. Of most importance are chronic infections with subclinical or mild to moderate clinical signs including low-grade anaemia, poor reproduction performance, and reduced growth rates [1, 11]. Both, acute and chronic *M. suis* infections have been reported worldwide causing significant economic losses to the pig industry [12–17].

Recently, a third porcine HM species was discovered in Zhejiang, China by Fu and co-workers in 2017 and designated as ‘*Candidatus* Mycoplasma haemosuis’ [18]. Phylogenetic analyses of the 16S rDNA revealed that this novel HM species is most closely related to the feline HM ‘*Candidatus* Mycoplasma turicensis’, the murine species *Mycoplasma muris* and the human HM ‘*Candidatus* Mycoplasma haemohominis’ [18]. This novel porcine HM species was also described in obviously healthy animals in Korea [19] and, most recently, in clinically affected fattening pigs in Germany with skin alterations, fever, and anaemia [20]. This clinical case indicated the need to establish a specific and sensitive detection method to investigate the spread of the novel emerging pathogen in the pig population.

In the last two decades, molecular detection methods for the so far uncultivable HMs have proven to be the basic prerequisite to get insights into the epidemiology, species, and strain differentiation and the clinical impact of HM infections [14, 21–26].

Thus, the aims of the present study were (i) to develop a specific real-time PCR diagnostic assay (qPCR) for the detection and quantification of ‘*Ca*. M. haemosuis’ in pigs and (ii) to apply this novel qPCR and investigate the occurrence of ‘*Ca*. M. haemosuis’ in fattening pigs as well as in sows and piglets in Germany. Because the piglets were sampled before the first colostrum uptake, additional information on a possible vertical transmission from dams to their offspring was obtained.

## Results

### Development of a real-time PCR assay

We developed a real-time qPCR assay for the specific detection of ‘*Ca*. M. haemosuis’ from the blood of pigs. The real-time qPCR is targeting the *gap* gene encoding the NAD-dependent glyceraldehyde 3-phosphate dehydrogenase of ‘*Ca*. M. haemosuis’ (GAPDH). For the establishment of the qPCR ‘*Ca*. M. haemosuis’ positive samples were available from a previous study [20]. In this study, pigs (n = 7) suffering from anaemia, fever, and skin alterations, were tested ‘*Ca*. M. haemosuis’-positive by 16S rDNA PCR, sequencing, and subsequent sequence analysis [20]. Our novel qPCR assay successfully detected ‘*Ca*. M. haemosuis’ in all blood samples from the ‘*Ca*. M. haemosuis’-infected pigs. Melting curve analyses revealed a specific melting temperature of 74.676 °C (± 0.32 °C). Moreover, we tested the specificity of the assay using bacterial strains and isolates as listed in Table 3. Positive qPCR reactions were found for ‘*Ca*. M. haemosuis’ DNA. No cross-reactivity was detected when DNA from non-target *Mycoplasma* spp. and other bacterial species representing 10^5^ bacteria/reaction were used as a template.

Sequence analyses of the qPCR amplicons from all available ‘*Ca.* M. haemosuis’ isolates (n = 7; 20) revealed 100% identity among each other and to the *gap* gene deposited in GenBank (Accession No. KU246051). The analytical sensitivity of the qPCR assay was determined using serial dilutions of the plasmid pC_*CMhsuis* standard corresponding to 10^7^ to 100 GE. The highest dilution yielding consistently positive qPCR results (Ct < 35 cycles) contained 0.45 fg pC_*CMhsuis* DNA per reaction corresponding to 10 gene copies per reaction. The lower limit of detection of the qPCR was thus 10 *gap* gene copies per reaction and 2.5 x 10^3^ genome equivalents per mL blood.

Quantitative PCR data analysis revealed a linear regression curve between 45 pg and 0.45 fg of the plasmid DNA. The PCR efficiency was calculated to be 97.3%. The intra-assay and inter-assay repeatability are shown in Table 1. All different concentrations in the standard dilutions from 10^7^ to 10^1^ GE were consistently detected by the qPCR assay.

**Table 1:** Inter-assay and intra-assay repeatability of the ‘*Ca*. M. haemosuis’ qPCR

	inter-assay repeatability (15 runs)	intra- assay repeatability (5 runs)
Copy number^a^	mean Ct^b^ (±SD)	mean Ct (±SD)
10^7^	15.27 (± 0.27)	15.31 (± 0.15)
10^6^	17.61 (± 0.82)	17.67 (± 0.13)
10^5^	21.08 (± 0.60)	21.33 (± 0.10)
10^4^	24.62 (± 0.64)	24.80 (± 0.10)
10^3^	27.68 (± 0.82)	27.69 (± 0.09)
10^2^	30.79 (± 0.70)	30.84 (± 0.15)
10^1^	33.51 (± 0.75)	33.52 (± 0.14)

^a^Copy numbers (GE/reaction) of the plasmid pC_*CMhsuis* were calculated from spectrophotometrically quantified DNA as described in Methods

^b^Mean threshold cycle values and standard deviations

Quantification of ‘*Ca*. M. haemosuis’ in the infected pigs [20] revealed blood loads from 3.08 × 10^2^ to 3.96 × 10^7^ bacteria/mL blood.

The ten randomly selected qPCR positive samples also revealed positive results in haemotrophic mycoplasma-specific16S rDNA amplification and sequencing. As all obtained sequences were identical, we uploaded one sequence to the GenBank (Accession No. MZ614253).

### ‘*Candidatus* M. haemosuis’ infections in fattening pigs

To investigate the occurrence of ‘*Ca*. M. haemosuis’ in fattening pigs, we tested a total of 200 animals from 20 fattening farms at the time of slaughter using the novel qPCR assay. ‘*Candidatus*. M. haemosuis’ was detected in blood samples of 35 out of 200 investigated animals (17.50%) originating from 9 out of the 20 farms (45.00%). Quantification of bacterial loads in qPCR-positive pigs revealed a mean value of 1.61 x 10^5^ ‘*Ca*. M. haemosuis’/mL blood (range: 5.52 x 10^3^ to 1.55 x 10^6^ ‘*Ca*. M. haemosuis’/mL). A total of 12 ‘*Ca*. M. haemosuis’ positive fattening pigs were co-infected with *M. suis* (Table 2). Data of all animals are included in Supplementary File 1.

**Table 2:** Occurrence of ‘*Ca*. M. haemosuis’ and *M. suis* in farms and on single animal level

	**piglet producing farms**	
	**‘*Ca*. M. haemosuis’**^**b**^	***M. suis***^**a**^
positive farms	3/21 (14.28%)	16/21 (76.19%)
farms with co-infections		3/21 (14.28%)
positive sows	13/208 (6.25%)	65/208 (31.25%)
positive piglets	28/622 (4.50%)	68/474 (14.35%)
born from positive sows	28	50
born from negative sows	0	18
co-infected sows		13/208 (6.25%)
co-infected piglets		4/474 (0.84%)
	**fattening farms**	
	**‘*Ca*. M. haemosuis’**	***M. suis***
positive farms	9/20 (45.0%)	10/20 (50.0%)
farms with co-infections		8/20 (40.0%)
positive pigs	35/200 (17.5%)	38/200 (19.9%)
co-infected pigs		12/200 (6.0%)

^a^In the study of Stadler and co-workers [17]

^b^This study

### ‘*Candidatus* M. haemosuis’ infections in farrowing sows and pre-suckling piglets

As shown in Table 2, a total of 13 out of 208 sows (6.25%) were positive for ‘*Ca*. M. haemosuis’ and the number of positive sows within a herd varied between one and ten. All 13 ‘*Ca*. M. haemosuis’ positive sows were co-infected with *M. suis*. On-farm level, ‘*Ca*. M. haemosuis’ was detected in three out of 21 investigated piglet-producing farms (14.29%) in at least one animal, whereas in the remaining 18 farms all animals were qPCR negative. In all farms, no clinical signs of IAP were obvious at the time of the investigation.

A total of 28 out of the 622 (4.50%) pre-suckling piglets reacted ‘*Ca*. M. haemosuis’ qPCR positive. All ‘*Ca*. M. haemosuis’ infected piglets originating from one farm and were born from 10 (76.92%) of the 13 qPCR positive sows. The number of positive piglets per sow varied between one and three. Four out of the 28 ‘*Ca*. M. haemosuis’ positive piglets were co-infected with *M. suis*.

Quantification of bacterial loads in qPCR-positive sows revealed a mean value of 3.83 x 10^4^ ‘*Ca*. M. haemosuis’/mL blood (range: 3.21 x 10^4^ to 6.44 x 10^4^ ‘*Ca*. M. haemosuis’/mL) and in qPCR-positive piglets a mean value of 2.25 x 10^5^ ‘*Ca*. M. haemosuis’/mL blood (range: 1.13 x 10^4^ to 2.48 x 10^6^ ‘*Ca*. M. haemosuis’/mL), respectively. Data of all animals are further included in Supplementary File 1.

## Discussion

In this study, we describe the establishment of a novel quantitative real-time PCR for the specific detection of the emerging porcine HM species ‘*Ca*. M. haemosuis’ in blood samples as well as the quantitative detection of ‘*Ca*. M. haemosuis’ in sows, piglets and fattening pigs from Germany. To our knowledge, our SYBR® green qPCR assay is the first quantitative diagnostic tool specific for ‘*Ca*. M. haemosuis’. The novel qPCR assay is targeting the ‘*Ca*. M. haemosuis’ gene encoding the NAD-dependent glyceraldehyde-3-phosphate dehydrogenase. The *gap* was chosen over the 16S rRNA gene because of an identity of >97% of the 16S rRNA gene of *Ca*. M haemosuis to other hemotrophic mycoplasmas (e. g. ‘*Ca*. M. turicensis’, ‘*Ca*. M. haemobos’, ‘*Ca*. M. haemomuris’, *M. haemocanis*, and *M. haemofelis*), Glyceraldehyde-3-phosphate dehydrogenase encoding genes were used as targets in -specific qPCR assays for the detection of haemotrophic and non-haemotrophic Mycoplasma species including *M. suis*, *M. wenyonii,* ‘*Ca*. M. haemobos’, *M. genitalium* and *M. hominis* [28, 29]. All these PCR assays have proven to be reliable and robust for use in prevalence studies and routine diagnostics [13, 17, 26, 30].

So far, ‘*Ca*. M. haemosuis’ was detected using 16S rDNA targeting primers without quantification of bacterial loads in infected pigs [18, 19]. The assay described herein allows quantification with a sensitivity of 10 genome equivalent per PCR corresponding to 2.5 x 10^3^ bacteria per mL blood. Comparable results have been obtained from previously published qPCR assays for the detection of other HMs, e.g. *M. suis*, the canine HM species *M. haemocanis*, ‘*Ca*. M. haematoparvum’ as well as the bovine HM species *M. wenyonii* and ‘*Ca*. M. haemobos’ [23, 26, 31]. This high analytical sensitivity enables the identification of asymptomatic chronically infected carrier animals that may serve as important epidemiological reservoirs [24, 32]. The analytical specificity of the novel ‘*Ca*. M. haemosuis’ qPCR assay as predicted *in silico* was confirmed by the negative PCR results obtained with all tested *Mycoplasma* spp. (HMs and non-haemotrophic mycoplasmas) and other porcine pathogens.

Reports about ‘*Ca*. M. haemosuis’ infections are scarce because the agent was recently discovered in 2017 [18]. In the study reported herein, we surveyed for the first time a European sample panel (n = 1080 pigs) for ‘*Ca*. M. haemosuis’ infections. We confirmed that ‘*Ca*. M. haemosuis’ is prevalent in Germany. Interestingly, we found considerable variation in the prevalence depending on the population studied. The highest detection rate with 17.5% was found in fattening pigs, followed by 6.25% in sows, and by 4.50% in piglets. In the two previous studies, the ‘*Ca*. M. haemosuis’ prevalence was higher in China with 36% positive sows and 24.1% positive growing pigs [18] and lower in Korea with only one ‘*Ca*. M. haemosuis’ positive animal (0.01%) out of 1867 tested pigs from 464 farms [19]. On-farm level, the higher detection rates for fattening pigs could be confirmed: only 3 out of the 21 piglet producing farms (14.29%) but 9 out of the 20 fattening farms (45.00%) were shown to be ‘*Ca*. M. haemosuis’ positive. Various factors could be responsible for the differences in the detection rates found between piglet producing and fattening farms including the purchase of pigs from different piglet-producing farms and potential higher biosecurity levels in piglet producing farms. In the current study, all positive sows were co-infected with ‘*Ca*. M. haemosuis’ and *M. suis*, but only 4 out of 28 piglets, and 12 out of 200 slaughter pigs were positive for both porcine HM species. The evidence of co-infections is in line with other HM studies. Co-infections with two or three HM species were also found in sows (26.7%) and growing pigs (13.9%) in China [18], in cattle [25, 26], in sheep [33], and cats [34].

So far, our knowledge regarding the pathogenicity of ‘*Ca*. M. haemosuis’ is rather limited. In the Chinese study, the novel porcine HM species was first detected in one diseased pig showing typical clinical signs of IAP [18]. In Europe, Stadler and co-workers (2020) identified ‘*Ca*. M. haemosuis’ first in diseased pigs showing also typical clinical signs of an *M. suis* induced IAP (i.e. anaemia, fever, skin alterations) [20]. In the present study ‘*Ca*. M. haemosuis’ was detected in obviously healthy pigs as it was also shown for *M. suis* in recent studies [13, 17, 35]. Typically, such chronic HM infections predominate in the pig population causing significant economic loss and welfare concern due to immune dysregulation, higher susceptibility to other infectious agents, extended feeding periods or increased stillbirth rates [4, 13, 17, 35, 36]. In addition, chronic HM infections can lead to increased and metaphylactic antibiotic usage contributing to the development of antibiotic resistance [36]. Further studies focusing on individual health parameters of ‘*Ca*. M. haemosuis’ positive pigs are certainly needed.

The ‘*Ca*. M. haemosuis’ loads found in the present study seem to be lower than those found for *M. suis* in sows (mean blood load of 3.15 × 10^7^ *M. suis*/mL [17];), in pre-suckling piglets (mean loads of 5.09 × 10^7^ *M. suis*/mL blood) or in fattening pigs (mean loads of 7.62 × 10^7^ *M. suis*/mL blood [13];, respectively. Interestingly, 76.92% of the positive sows have born at least one ‘*Ca*. M. haemosuis’ positive piglet indicating that ‘*Ca*. M. haemosuis’ is transmitted vertically within the pig herds. The possibility of vertical transmission of HMs has also been described for *M. suis* [17] as well as for *M. wenyonii* and ‘*Ca*. M. haemobos’ [37, 38]. But it is worth noting when comparing the vertical transmission of *M. suis* [17] and ‘*Ca*. M. haemosuis’ (present study) that a considerably higher percentage of 76.92% of the ‘*Ca*. M. haemosuis’ infected sows delivered infected pre-suckling piglets whereas only 50% of the *M. suis* infected sows have born positive piglets.

## Conclusion

We showed for the first time that ‘*Candidatus* Mycoplasma haemosuis’ infection is prevalent in Germany in piglet producing farms as well as in fattening farms and coinfections with *M. suis* are common. Our data on the detection of ‘*Ca*. M. haemosuis’ in pre-suckling piglets indicate that the pathogen is transmitted vertically. Further studies are needed to investigate the pathogenic potential, the clinical impact, prevalence, and epidemiology including transmission routes of ‘*Ca*. M. haemosuis’ to clarify the significance of this emerging pathogen. The herein-described novel qPCR assay can be used to accurately diagnose infections with the new HM species in pigs and to perform these studies.

## Methods

### Sample and data collection

Blood samples (n = 7) of ‘*Ca*. M. haemosuis’ positive fattening pigs were available from a previous study [20]. As samples were taken as diagnostic material during acute disease, no ethical approval was required according to the German Animal Welfare Law. The pigs suffering from skin alterations (urticaria, haemorrhagic diathesis), high fever and anaemia were shown to be ‘*Ca*. M. haemosuis’ positive by 16S rDNA amplification and subsequent sequence analysis [20]. The seven animals revealed a negative *M. suis*-qPCR result.

In addition, DNA samples extracted from the blood of farrowing sows (n = 208) and corresponding pre-suckling piglets (n = 622) from 21 piglet producing farms were available from a previous study [17]. From each farm, nine to ten farrowing sows and two or three pre-suckling piglets per sow have been sampled. DNA quality was checked using a NanoDrop™ 2000 to assure the quality of the samples. Animal sampling was performed in accordance with the German animal welfare law using a protocol officially approved by the Government (Az. 55.2–154–2532.2-16-13).

For the group of fattening pigs, EDTA-anticoagulated blood samples were taken from 200 animals (20 different farms in Germany, 10 animals per farm) at the time of slaughter. All 20 farms were located in the South of Germany (Federal States of Bavaria and Baden-Wuerttemberg). Blood collection was performed after slaughtering, thus, no ethical approval was needed for those samples according to the German Animal Welfare Law and the DIRECTIVE 2010/63/EU.

Bacterial DNA was extracted as described elsewhere [13, 17]. Briefly, blood samples were preconditioned by mixing 200 μl of EDTA-anticoagulated blood with an equal volume of lysis buffer (10 mM Tris pH 7.5, 5 mM MgCl2, 330 mM sucrose, 1% (v/v) Triton X-100). The mixure was centrifuged (8000 x g, 1 min, 20 °C) and the pellet was again washed twice with 400 μl lysis buffer. Subsequently, a total amount of 200 μl bacterial DNA was using the GenElute™ Bacterial Genomic DNA Kit (Sigma-Aldrich, Steinheim, Germany). One PBS control was included into each extraction run of ten blood samples. For all samples, the *M. suis* status was determined by a quantitative PCR [17, 23] either in a previous study (sows and piglets, [17]) or in the present study (fattening pigs).

### Primer design and amplicon sequencing

To develop a specific ‘*Ca*. M. haemosuis’ qPCR two primers were designed based on the gene *gap* encoding the NADP-dependent glyceraldehyde-3-phosphate dehydrogenase of ‘*Ca*. M. haemosuis’ (Accession No. KU246051) using the Primer3 software [39, 40]: *CMhsuis*F 5´-TGCTTTGGCTCCTGTGGTTA-3´ and *CMhsuis*R 5´- GCAGCAGCACCTGTAGAAGTA-3`. The Blast algorithm, which is provided by NCBI, was used to test the specificity of the primers *in silico*. Specificity was further investigated by sequencing (Seqlab Sequence Laboratories, Göttingen, Germany) of the resulting 177-bp *gap* fragment of ‘*Ca*. M. haemosuis’. Obtained sequences were compared to GenBank entries using the Blast tool provided by NCBI.

### Cloning and Preparation of Standard DNA

The 177-bp qPCR fragment of ‘*Ca*. M. haemosuis’ was cloned into the plasmid vector pCR2.1 (Invitrogen) according to the manufacturer’s instructions. Plasmid DNA was purified from the *Escherichia coli* transformant (pC_*CMhsuis*) using the GenElute™ Plasmid Miniprep Kit (Sigma-Aldrich). Plasmid DNA was quantified using a spectrophotometer (NanoDrop™ 2000, Thermo Fisher Scientific). DNA concentrations were adjusted to 45 pg/2 μL representing 1 x 10^7^ GE according to the calculation described below.

### Quantitative real-time PCR

‘*Candidatus*. M. haemosuis’ DNA was detected and quantified with the StepOne™ System (Applied Biosystems). The 20 μL reaction mixtures contained 10 μL of the 2x SYBR® Green PCR Master Mix (Thermo Fisher Scientific), 8 μL primer mixture (containing 0.5 μM primer each), and 2 μL template DNA. Cycling conditions consisted of 95 °C for 15 min and 40 cycles at 95 °C for 15 s and 60 °C for 1 min followed by a melting curve analysis. Plasmid pC_*CMhsuis* DNA standard dilutions (450 fg/2 μl, 45 fg/2 μl, and 4.5 fg/2 μl) representing 10^5^, 10^4^, and 10^3^ GE per reaction were included in each qPCR run for quantification. Obtained Ct values were extrapolated into ‘*Ca*. M. haemosuis’ GE/reaction using the Standard Curve Method of the StepOne™ Software Version 2.2 (Applied Biosystems). The ‘*Ca*. M. haemosuis’ GE/mL blood were determined considering the factor 200 (2 μL template out of 200 μL DNA volume out of 200 μL EDTA blood (software Microsoft® Excel, 2016).

Since the diagnostics of hemotrophic mycoplasmas in Germany has to be covered by the farmers themselves, we decided to establish a SYBR Green assay, to use the range of 10^5^–10^3^ GE/reaction and to omit an internal control. Despite some disadvantages compared to the use of probe-based PCRs, the SYBR Green assay offers a clear economic advantage with nevertheless good diagnostic specificity [41]. This allows us to keep costs as low as possible and to offer farmers an incentive to send in diagnostic samples. *Analytical specificity and lower limit of detection of the real-time qPCR.*

The specificity of the qPCR assay was tested by using template DNA from the porcine HM species *M. suis*, *M. parvum*, other haemotrophic and non-haemotrophic *Mycoplasma* spp. and a panel of other porcine pathogens (Table 3). Bacteria were cultivated and/or DNA was isolated as described elsewhere [17, 23, 26].

**Table 3:** Bacterial isolates for testing the specificity of the ‘*Ca*. M. haemosuis’ qPCR

Bacterial species	Origin
*‘Candidatus* M. haemosuis’ (n = 7)	[20], University of Hohenheim^a^ (DNA)
*Mycoplasma suis* KI82 (n = 4) *Mycoplasma parvum*	[27], University of Hohenheim^a^ (DNA) University Hohenheim^a^(DNA)
*Mycoplasma wenyonii* (n = 3)	[26], University of Hohenheim^a^ (DNA)
*‘Candidatus* M. haemobos’ (n = 3)	[26], University of Hohenheim^a^ (DNA)
*Mycoplasma haemofelis*	University Zurich^b^ (DNA)
*Mycoplasma fastidiosum*	ATCC 33229
*Mycoplasma hyorhinis*	ATCC 17981
*Mycoplasma hyosynoviae*	University Bern^c^ (DNA)
*Mycoplasma arginini*	University Bern^c^ (DNA)
*Mycoplasma dispar*	University Bern^c^ (DNA)
*Mycoplasma flocculare*	University Bern^c^ (DNA)
*Mycoplasma bovis (n = 3)*	University Bern^c^ (DNA)/ATCC 25523
*Mycoplasma bovirhinis*	ATCC 27748
*Mycoplasma bovigenitalium*	University Bern^c^ (DNA)
*Mycoplasma bovoculi*	University Bern^c^ (DNA)
*Mycoplasma capricoum spp. capricolum*	University Bern^c^ (DNA)
*Salmonella* Typhimurium (n = 2)	University Hohenheim^a^
*Staphylococcus aureus*	University Hohenheim^a^
*Escherichia coli* (n = 2)	University Hohenheim^a^
*Pasteurella multocida*	CVUA Stuttgart^d^
*Streptococcus suis*	ATCC 43765

^a^Institute of Animal Science, University Hohenheim, Stuttgart, Germany

^b^Institute of Veterinary Bacteriology, University Zurich, Switzerland

^c^Institute of Veterinary Bacteriology, University Bern, Switzerland

^d^Chemical and Veterinary Investigations Office, Stuttgart, Germany

For measuring the lower limit of detection (LOD) pC_*CMhsuis* plasmid DNA concentration was adjusted to 45 pg/2 μL representing 1 x 10^7^ GE/2 μL and the LOD was measured by testing ten-fold dilutions (from 10^7^ to 1 GE/reaction) in 15 runs.

The ‘*Ca*. M. haemosuis’ genome was calculated as 0.85 fg, and the pC_*CMhsuis* plasmid corresponds to 4.5 ag per copy (genome weight = genomic length (bp) x 10^5^ x 665 Da/bp x 1.67 x 10^24^ g/Da) (http://cels.uri.edu/gsc/cndna.html). Since the actual genome size of ‘*Ca*. M. haemosuis is unknown so far, we used a mean genome size of all so far sequenced haemotrophic mycoplasmas of 750 kb. For the plasmid, a size of 4.106 kb was used to calculate the concentrations in plasmid copies per microliter corresponding to genome equivalents (GE) of ‘*Ca*. M. haemosuis’.

Four replicates of the plasmid dilutions (10^7^ to 10^1^ GE/reaction) were tested in the same run to assess the intra-assay repeatability. The inter-assay repeatability was determined by running duplicates of the same plasmid dilutions in five different runs on different days carried out by two persons.

### Conventional PCR

For confirmation of the new developed qPCR, we tested a total out of ten randomly selected qPCR positive results by haemotrophic mycoplasma-specific16S rDNA PCR as described elsewhere [42]. The ten amplicons were sequenced (Seqlab Sequence Laboratories, Göttingen, Germany). Obtained sequences were compared to GenBank entries using the Blast tool provided by NCBI.

## Supplementary Information

**Additional file 1.**

## Data Availability

The datasets used and/or analysed during the current study are available from the corresponding author on reasonable request.

## Abbreviations

*Ca*.: *Candidatus*
IAP: Infectious anaemia in pigs
HM: Haemotrophic mycoplasma
*M.*: *Mycoplasma*
qPCR: Quantitative polymerase chain reaction
